# The G311E Mutant Gene of MATE Family Protein DTX6 Confers Diquat and Paraquat Resistance in Rice Without Yield or Nutritional Penalties

**DOI:** 10.3390/ijms26136204

**Published:** 2025-06-27

**Authors:** Gaoan Chen, Jiaying Han, Ziyan Sun, Mingming Zhao, Zihan Zhang, Shuo An, Muyu Shi, Jinxiao Yang, Xiaochun Ge

**Affiliations:** 1 State Key Laboratory of Genetics and Development of Complex Phenotypes, School of Life Sciences, Fudan University, 2005 Songhu Road, Shanghai 200438, China; 22210700103@m.fudan.edu.cn (G.C.); 24210700018@m.fudan.edu.cn (J.H.); 21110700074@m.fudan.edu.cn (Z.S.); 19210700173@fudan.edu.cn (M.Z.); 23210700082@m.fudan.edu.cn (Z.Z.); 23110700001@m.fudan.edu.cn (S.A.); 24110700069@m.fudan.edu.cn (M.S.); 2 Beijing Key Laboratory of Maize DNA Fingerprinting and Molecular Breeding, Beijing Academy of Agriculture and Forestry Sciences, Beijing 100097, China; yangjinxiao@maizedna.org

**Keywords:** weed control, DTX6m, herbicide, paraquat, diquat, yield, nutrition

## Abstract

Weeds present a pervasive challenge in agricultural fields. The integration of herbicide-resistant crops with chemical weed management offers an effective solution for sustainable weed control while reducing labor inputs, particularly in large-scale intensive farming systems. Consequently, the development of herbicide-resistant cultivars has emerged as an urgent priority. In this study, we found that the G311E mutant gene of *Arabidopsis* MATE (multidrug and toxic compound extrusion) family transporter DTX6, designated DTX6m, confers robust resistance to bipyridyl herbicides paraquat and diquat in rice. *DTX6m*-overexpression lines exhibited marked resistance to these two herbicides, tolerating diquat concentrations up to 5 g/L, which is five-fold higher than the recommended field application dosage. Agronomic assessments demonstrated that grain yields of *DTX6m*-overexpressing plants were statistically equivalent to those of wild-type plants. Moreover, the plants displayed beneficial phenotypic changes, such as accelerated flowering and a slight reduction in height. Seed morphometric analysis indicated that in comparison with the wild-type control, *DTX6m*-transgenic lines exhibited altered grain dimensions while maintaining consistent 1000-grain weight. Nutritional assays further demonstrated that DTX6m increased the levels of free amino acids in seeds, while normal protein and starch contents were retained. Collectively, these results establish that *DTX6m* effectively boosts rice resistance to paraquat and diquat, validating DTX6m as a candidate gene for engineering plant herbicide resistance and also implying a potential role for *DTX6m* in amino acid homeostasis in plants.

## 1. Introduction

Weeds pose a major threat to agricultural productivity in cultivated areas. They compete with crops for essential resources such as water, nutrients, and sunlight, leading to decreased yields and overall agricultural output [1]. Traditional manual weeding methods, though effective, are highly labor-intensive and becoming increasingly unsustainable due to rural labor shortage and declining birth rates [1,2]. As a result, chemical herbicides have become the dominant weed control method in modern agriculture, offering both high efficacy and cost efficiency [3]. Large-scale cultivation of major food crops inherently requires herbicide-resistant traits. This imperative is particularly pronounced for rice, a staple crop feeding over half of the global population. Weed infestation threatens rice yields, a challenge further exacerbated by the transition from conventional transplanting to modern direct-seeding cultivation systems [4,5]. Herbicide application, combined with herbicide-resistant rice varieties, offers an effective weed management solution with minimal labor demands, making it indispensable for large-scale rice production [6].

The identification of herbicide-resistance genes is crucial for developing herbicide-resistant crops. By isolating these genes, which often are derived from resistant weeds, bacteria, or other plant species, researchers can employ genetic engineering to introduce herbicide tolerance into crops, ultimately producing elite cultivars [7,8]. Through the introduction and upregulation of genes responsible for herbicide detoxification, scientists can enhance the herbicide resistance of crops without negatively affecting their agronomic performance or yield [8,9].

To date, numerous synthetic herbicides with diverse selectivity and modes of action have been commercialized and widely adopted [10,11]. Developing crop cultivars resistant to various herbicides and implementing rotations of crops with different herbicide resistances are essential strategies to mitigate the risk of evolved weed resistance, which often results from the overreliance on single herbicide-resistant crops [2,12]. Diquat, a bipyridyl herbicide with structural similarity to paraquat, exerts its phytotoxic effects by accepting electrons from chloroplast photosystem I (PSI) under light conditions. The subsequent transfer of these electrons to molecular oxygen generates reactive oxygen species, inducing severe oxidative stress and leading to rapid plant tissue necrosis [13,14]. Compared with paraquat, diquat maintains comparable herbicidal efficacy but exhibits lower mammalian toxicity. In recent years, diquat has been widely used to replace paraquat in many countries [15,16,17]. Additionally, diquat can be absorbed by soil particles and undergoes degradation, resulting in reduced environmental persistence and enhanced ecological safety [13].

In our previous study, we identified a bipyridyl herbicide-resistance gene named *DTX6* in *Arabidopsis* [18]. This gene encodes a MATE (multidrug and toxic compound extrusion) family transporter. A glycine-to-glutamic acid substitution at position 311 (G311E) confers enhanced paraquat resistance in *Arabidopsis* [18,19]. DTX6 comprises 12 transmembrane domains with dual localization to both vacuolar and plasma membranes. Though its in vivo substrate remains undetermined, it mediates paraquat and diquat detoxification through vacuolar sequestration and exocytotic extrusion [18]. While these findings establish the molecular basis of resistance in dicots, whether this mutation confers cross-species resistance or induces pleiotropic effects remains unknown. In this study, we demonstrate that the G311E-mutated DTX6 (designated DTX6m) confers robust resistance to paraquat and diquat in rice. Furthermore, DTX6m induces mild dwarfism and accelerates flowering time without compromising grain yield, while significantly increasing seed amino acid content. These results highlight its agricultural potential for developing herbicide-resistant crops through genetic breeding without sacrificing agronomic performance and also provide mechanistic insights for investigating its physiological substrates.

## 2. Results

### 2.1. Generation of DTX6m-Expressing Rice Lines

**Figure 1:**
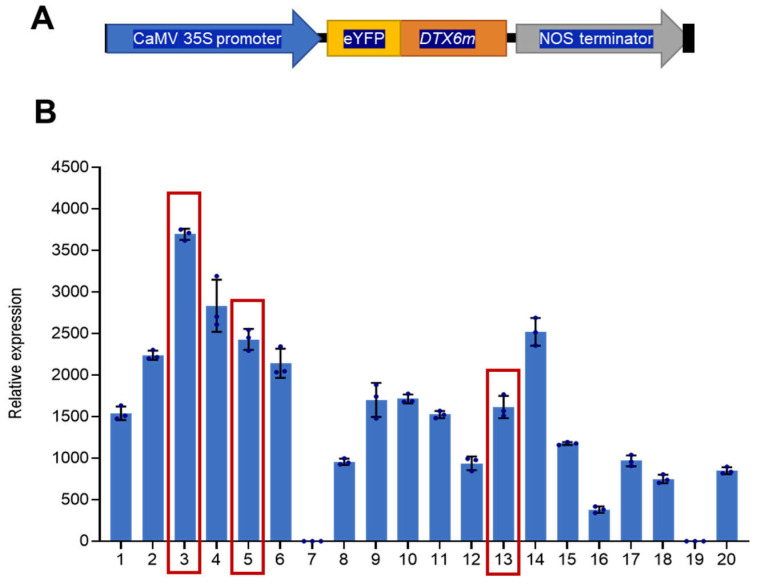
Generation of DTX6m-overexpression lines. (**A**) Schematic diagram of the *35S::eYFP-DTX6m* vector; (**B**) Identification of the *DTX6m*-overexpression lines by RT-qPCR. RNA was extracted from leaves of two-week old transgenic seedlings and then used for the examination. The expression level of *DTX6m* relative to *UBQ5* is shown. Data represent means ± SD of three technical replicates. Scale bar = 10 cm.

The G311E-mutated *DTX6* allele, *DTX6m*, was cloned into the pCAMBIA-N-eYFP vector under the promoter of CaMV 35S and transformed into the rice cultivar ZH11 (Figure 1A), considering that ZH11 is widely used in rice research and its genome has been fully sequenced. Screening of T_0_ transgenic plants identified 20 independent lines harboring the *DTX6m* transgene, which were designated OE1 to OE20. Quantitative RT-PCR analysis revealed substantial variation in *DTX6m* expression level across these lines (Figure 1B), with the majority exhibiting significant transgene expression. T-DNA insertion at different genomic loci may result in differential transgene expression, as the genomic context of the insertion site can influence transgene activity. Moreover, insertional events may disrupt endogenous gene function during transgene expression. To evaluate the correlation between expression levels and herbicide resistance and mitigate potential impacts of endogenous gene disruption, three representative lines were chosen for phenotypic characterization: OE-3 (highest level), OE-5 (intermediate level), and OE-13 (relatively low level).

### 2.2. DTX6m-Overexpression Lines Exhibit Strong Paraquat and Diquat Resistance

**Figure 2:**
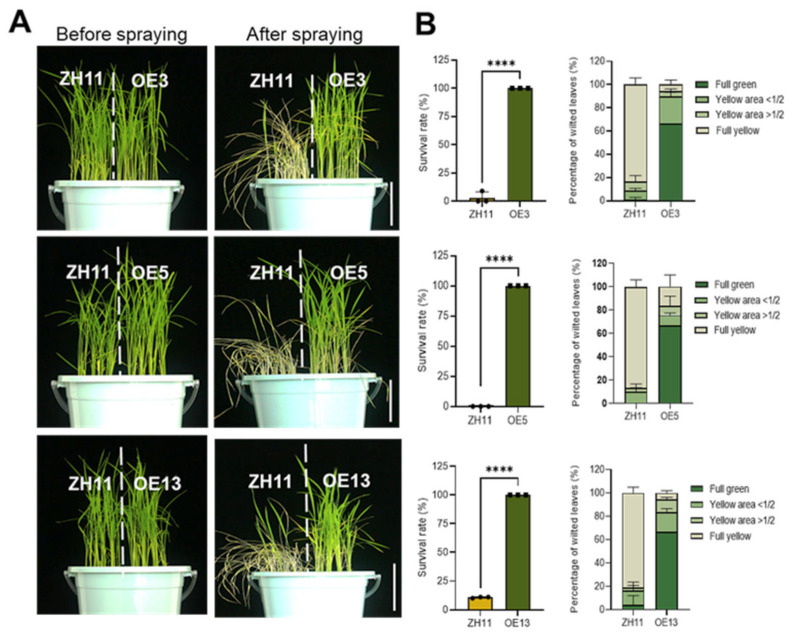
Paraquat resistance phenotypes of *DTX6m*-overexpression lines. (**A**) Growth conditions of plants before and after spraying with paraquat. ZH11 and three *DTX6m*-overexpression lines were grown in soil for one month and then sprayed with 0.5 g/L paraquat. Plants were photographed 10 days after spraying. (**B**) The survival rate and wilted leaves were documented. Data represent means ± SD of three biological replicates. Each replicate includes 10 rice plants. One-way ANOVA was performed to analyze the significance of difference between ZH11 and *DTXm*-overexpression lines. **** *p* ≤ 0.0001.

To evaluate resistance to bipyridyl herbicides, we utilized the T3 generation of the representative lines whose homozygosity were confirmed by the absence of segregation in hygromycin resistance. To preliminarily observe the herbicide resistance on culture media, OE3, 5, 13, and the ZH11 control were grown on 1/2 MS media supplemented with 0.3 µM paraquat for 8 days. As shown in Figure S1, all overexpression lines had strikingly higher seedling heights and longer primary roots compared with the wild-type control, which were severely stunted by the herbicide in the media. To compare their resistance to foliar-applicated herbicide, seedlings grown in soil for 25 days were foliar-sprayed with 0.5 g/L paraquat. Ten days after application, leaves of the ZH11 wild type exhibited severe necrosis, whereas all three overexpression lines maintained green foliage with minimal phytotoxicity symptoms (Figure 2A). Quantitative analysis confirmed significantly higher survival rates and more retention of functional green leaves in transgenic lines compared with the wild type (Figure 2B). These findings conclusively demonstrate that constitutive expression of *DTX6m* confers substantial paraquat tolerance in rice.

**Figure 3:**
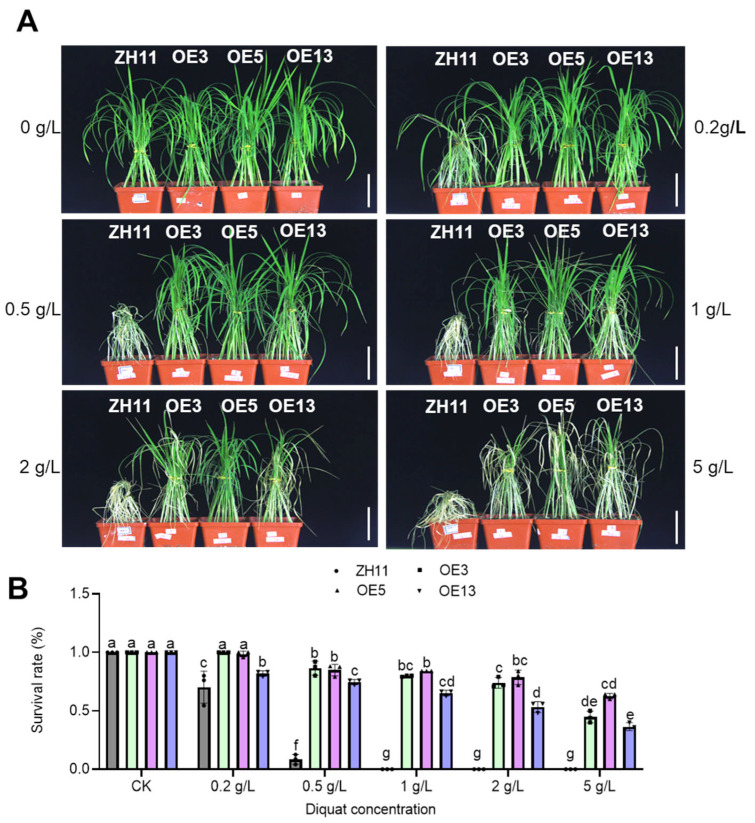
Evaluation of the resistance of the *DTX6m*-transgenic lines to different concentrations of diquat. (**A**) Diquat resistance phenotypes of ZH11 and *DTX6m*-transgenic lines. ZH11 and three *DTX6m*-overexpression lines were grown in soil for 25 days and then sprayed with diquat at different concentrations (0, 0.5, 1, 2, and 5 g/L). Plants were photographed 3 days after herbicide application. (**B**) The survival rates of ZH11 and *DTX6m*-transgenic lines. Data represent means ± SD of three biological replicates. Each replicate includes 20 rice plants. Two-way ANOVA was performed to analyze the significance of difference, with Fisher’s LSD test. Scale bar = 5 cm.

**Figure 4:**
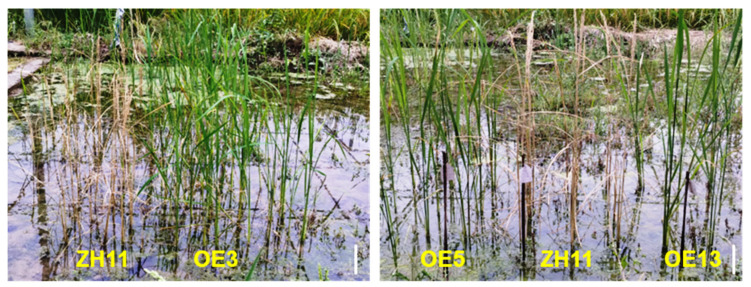
Diquat resistance phenotypes of three *DTX6m*-overexpression lines in the field. Rice plants were grown in the field for two months and then sprayed with 2 g/L diquat. Each transgenic line was represented by 16 plants. Pictures were taken 10 days after spraying. Scale bar = 10 cm.

Given the prevalent agricultural usage of diquat as an alternative bipyridyl herbicide to paraquat in many countries, we evaluated diquat resistance in rice plants through multi-stage physiological assessments. Initial screening at the seedling stage employed 25-day-old soil-grown plants subjected to foliar application of diquat at escalating concentrations (0, 0.5, 1, 2, and 5 g/L). Post-treatment evaluation after three days revealed a concentration-dependent response. ZH11 plants exhibited severe chlorosis at all tested concentrations, whereas transgenic lines maintained significant chlorophyll integrity even at 5 g/L (5 × the commercial field application rate) (Figure 3A). Quantitative analysis demonstrated differential tolerance among transgenic lines, with OE3 and OE5 showing significantly stronger resistance compared with OE13 (Figure 3B), correlating with the higher expression levels of *DTX6m* in OE3 and OE5 (Figure 1). Field validation experiments involved diquat application (2 g/L) at the reproductive stage (2 months post-transplant in the paddy field). Ten days after treatment, ZH11 plants displayed complete systemic necrosis and mortality, while all transgenic lines survived without chlorosis (Figure 4). This translational evidence confirms that *DTX6m* overexpression confers robust cross-environmental tolerance to diquat.

### 2.3. DTX6m-Overexpression Lines Had Early Flowering and Semi-Dwarf Growth Phenotypes in the Field

**Figure 5:**
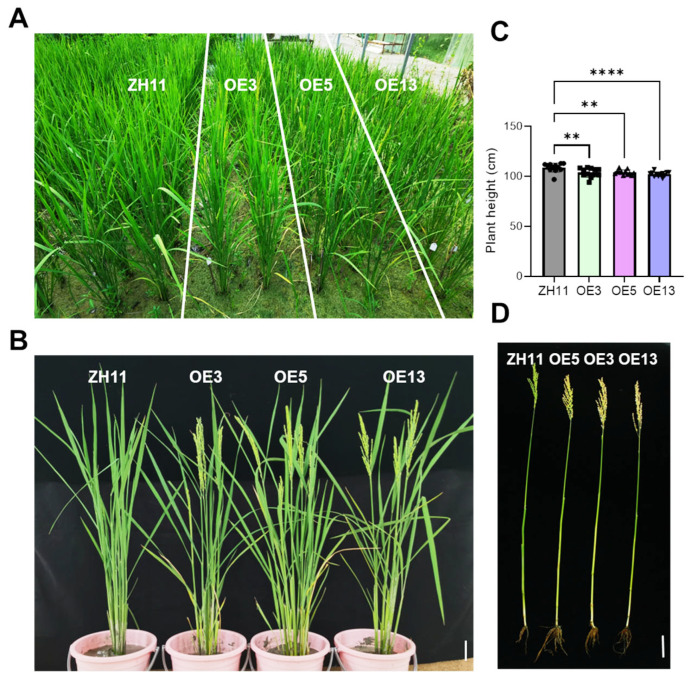
Phenotypes of the transgenic lines in the field. (**A**) Flowering rice plants in the field. (**B**) Phenotype comparison of ZH11 and three *DTX6m*-overexpression lines. (**C**) Comparison of plant height in the field. Data represent means ± SD (n = 12 plants). One-way ANOVA was performed to analyze the significance of difference between ZH11 and different *DTXm*-overexpression lines. **** *p* ≤ 0.0001. ** *p* ≤ 0.01. (**D**) Phenotype of the main tiller at day 110 after breeding. Scale bar = 10 cm.

To systematically characterize the possible pleiotropic effects of *DTX6m-* overexpression in rice, we conducted side-by-side field trials of transgenic lines and ZH11 wild-type controls. While vegetative growth showed no significant differences between the groups, pronounced phenotypic variations emerged during maturation. The *DTX6m*-overexpressing lines displayed reduced plant height and approximately 3–4 days earlier flowering, accompanied by earlier leaf senescence (Figure 5A–C). Analysis of primary tillers revealed that transgenic lines produced significantly shorter tillers with precocious maturation compared with ZH11 (Figure 5D). These findings demonstrate that *DTX6m* also functions as a developmental regulator in rice, in addition to its herbicide-detoxifying capacity.

### 2.4. Analysis of the Field Agronomic Traits of DTX6m-Transgenic Lines

**Figure 6:**
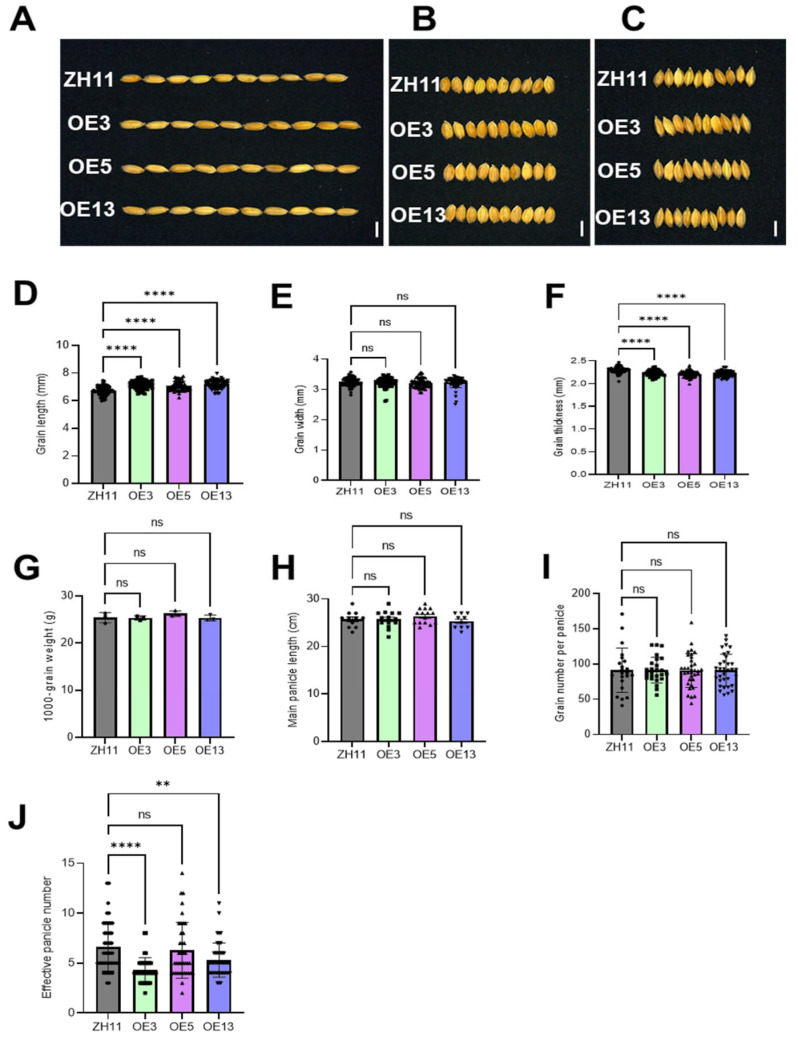
Comparative analyses of the grain size and yield traits of ZH11 and *DTX6m*-overexpression lines. (**A**) Grain length. (**B**) Grain width. (**C**) Grain thickness. (**D**) Statistical analysis of the grain length. (**E**) Statistical analysis of the grain width. (**F**) Statistical analysis of the grain thickness. In (**D**–**F**) Data represent means ± SD (n = 65 seeds from different plants). (**G**) Statistical analysis of 1000-grain weight (n = 3). (**H**) Statistical analysis of the main panicle length (n ≥ 10). (**I**) Statistical analysis of the grain number per panicle (n ≥ 22). (**J**) Statistical analysis of the effective panicle number (n ≥ 38). One-way ANOVA was used to analyze the significance of difference between ZH11 and different *DTXm*-overexpression lines. **** *p* ≤ 0.0001. ** *p* ≤ 0.01. In (**A**–**C**), Scale bar = 5 mm.

Yield traits are essential considerations in the application of genetically modified crops. We therefore evaluated the impact of *DTX6m* overexpression on agronomic traits associated with grain yield, including grain size, 1000-grain weight, number of effective panicles per plant, panicle length, and grain number per panicle. As shown in Figure 6A–F, OE3, OE5, and OE13 lines displayed three distinct morphological characteristics: (1) increased grain length, (2) comparable grain width, and (3) a slight reduction in grain thickness compared with ZH11. Statistical analysis revealed no significant differences in 1000-grain weight between *DTX6m*-OE lines and ZH11 (Figure 6G). Evaluation of the panicle architecture showed consistent patterns in both the main panicle length and grain numbers per panicle across all lines (Figure 6H,I). However, phenotypic divergence emerged in terms of effective panicle numbers, with OE3 and OE13 showing significant reductions relative to ZH11, while OE5 maintained wild-type levels (Figure 6J). This differential performance suggests that field-effective panicle formation exhibits environmental sensitivity, with the OE5 line demonstrating better field adaptability, which is likely to be attributable to the preserved tillering capacity compared with other transgenic lines.

### 2.5. Seed Storage Reserve Accumulation Is Not Consistently Affected in the Transgenic Lines, Except the Free Amino Acid Level

**Figure 7:**
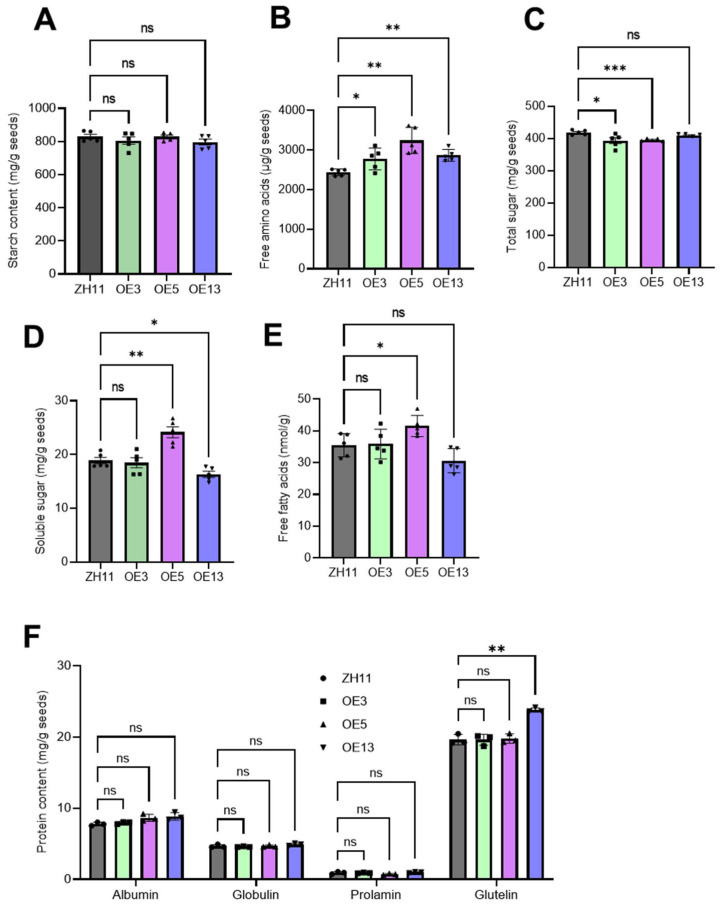
A comparative analysis of the nutritional components of seeds from ZH11 and *DTX6m*-overexpression lines. (**A**) Free fatty acid levels. (**B**) Free amino acid levels. (**C**) Total sugar. (**D**) Soluble sugar. (**E**) Starch content. (**F**) Albumin, globulin, prolamine, and glutelin levels. Each determination was performed with 5 replicates. One-way ANOVA was used to analyze the significance of difference between ZH11 and *DTX6m*-overexpression lines. *** *p* ≤ 0.001. ** *p* ≤ 0.01. * *p* ≤ 0.1.

Rice seeds, as a staple food, serve as a source of carbohydrates, proteins, and lipids for human consumption. To determine whether the overexpression of *DTX6m* altered the nutritional quality of rice seeds, we analyzed the levels of starch, total sugar, soluble sugars, free lipidic acids, free amino acids, and proteins in transgenic rice lines. The results revealed that the transformation of *DTX6m* did not change the starch content (Figure 7A), but did elevate the free amino acid levels in all three transgenic lines (Figure 7B). Regarding the total sugar, soluble sugar, and free lipidic acid levels, there were no consistent changes in the three transgenic lines (Figure 7C–E), suggesting that their content might have been affected by factors other than *DTX6m*. The protein content, including albumin, globulin, prolamin, and glutelin levels, remained unchanged in the transgenic lines (Figure 7F). Among the three *DTX6m*-overexpression lines, OE5 performed exceptionally well (Figure 7A–E), exhibiting significantly higher levels of free fatty acids, free amino acids, and soluble sugar compared with ZH11.

## 3. Discussion

### 3.1. Evolutionary Analysis of Arabidopsis and Rice MATE Family Genes

MATE family proteins, belonging to a large superfamily of evolutionarily conserved membrane transporters, are widely distributed across diverse organisms ranging from bacteria to plants and animals [20]. In plants, these proteins exhibit distinct subcellular localizations, including the plasma membrane [21,22], tonoplast [23,24,25,26], Golgi apparatus [27], and late endosome/prevacuolar compartment [28].They mediate a wide spectrum of physiological processes such as heavy metal and herbicide detoxification [18,19,21]; aluminum tolerance [29,30]; iron homeostasis regulation [22,27,31]; vacuolar sequestration of secondary metabolites [23,24,26,32]; hormone delivery [33,34,35,36]; leaf senescence and turgor modulation [25,28]; and pathogen resistance [37,38,39]. Notably, the substrate specificity of MATE family members shows remarkable diversity, necessitating individual characterization for each transporter.

The MATE transporter family comprises 56 members in *Arabidopsis thaliana* [40] and 46 members in rice (*Oryza sativa*) [41]. Phylogenetic analysis of MATE proteins from both species revealed that the rice homologs LOC_Os01g31980, LOC_Os01g49120, and LOC_Os05g48040 showed the closest evolutionary proximity to *Arabidopsis* DTX6 (Figure S2). Although clustered within the same phylogenetic subgroup, these proteins occupy distinct branches with relatively low sequence identities to DTX6 (55.8%, 53.9% and 50.7%, respectively), highlighting substantial divergence of MATE transporters between monocots and dicots. To functionally explore their relationship, we employed single-base editing technique [42] to introduce a G311E substitution (equivalent to DTX6’s critical glycine residue) in LOC_Os01g31980 (Figure S3). Unexpectedly, the edited rice plants exhibited no phenotypes with altered resistance to paraquat or diquat herbicides, suggesting that LOC_Os01g31980 may not serve as the functional ortholog of DTX6 in rice. We performed in silico subcellular localization predictions for the three rice DTX6 homologs using the WoLF PSORT algorithm (https://wolfpsort.hgc.jp, accessed on 25 June 2025). The results indicated that all three proteins were predicted to localize to the plasma membrane, whereas our previous data revealed that DTX6 localizes to both the plasma membrane and tonoplast. This distinct subcellular localization pattern may contribute to the phenotypic divergence observed between *DTX6* and *LOC_Os01g31980* mutants, in addition to potential substrate-specific differences.

### 3.2. DTX6m Largely Increased the Resistance of Rice to Paraquat and Diquat

Weed infestation poses a significant threat to global rice production, with yield losses exceeding 40% in severe cases [8]. In large-scale rice cultivation, the integration of herbicide-resistant rice cultivars with compatible herbicides has emerged as an effective strategy for weed control. Recent advances in understanding plant–herbicide interactions have driven the development of herbicide-tolerant rice through three principal strategies including traditional mutagenesis and phenotypic screening, precise editing of endogenous resistance genes, and transgenic introduction of exogenous resistance determinants [2,28]. In previous studies, we found that DTX6 mediates paraquat and diquat detoxification through exocytosis and vacuolar sequestration mechanisms. Notably, when glycine at position 311 in *Arabidopsis* DTX6 is substituted with acidic amino acid (G311E or G311D), the protein exhibits enhanced detoxification capacity [18]. In this study, we investigated the potential application of the *DTX6m* gene and its side effects in monocot crop rice. Three independent *DTX6m*-overexpression lines (OE3, OE5 and OE13) demonstrated remarkable bipyridyl herbicide resistance compared with the ZH11 wild type. Greenhouse trials with rice seedlings showed that while ZH11 plants completely wilted in 3 days after 1 g/L diquat treatment, all three transgenic lines maintained >80% survival rates. Even at 5 g/L diquat (5 × concentration), a significant number of the transgenic plants survived, with OE5 showing the strongest resistance (over 60%). Field trials conducted on two-month-old plants revealed complete mortality of ZH11 within 10 days after 2 g/L diquat application, whereas all transgenic lines survived, demonstrating stable, long-term diquat resistance that appears more effective in mature plants. These results suggest that DTX6m functions similarly in rice and *Arabidopsis*, probably through the same mechanisms.

### 3.3. The Transgenic Rice Had Slightly Early Flowering and a Dwarf Phenotype Without Compromised Yield and Nutritional Value

The potential pleiotropic effects of *DTX6*m transgenesis on plant growth, development, yield, and nutrient assimilation were systematically investigated. In the field trials, all three transgenic lines exhibited two phenotypes with significant agricultural value: slightly early flowering and semi-dwarfism. Early flowering shortens the growth cycle, optimizing land use efficiency. Meanwhile, the semi-dwarf trait enhances lodging resistance, which is particularly advantageous in typhoon-prone regions.

Before implementing *DTX6m* in genetic manipulation, it is crucial to determine its impact on crop yield and grain quality. Therefore, we evaluated key rice yield components, including the number of panicles per plant, the number of grains per panicle, and 1000-grain weight. The results showed that the OE3 and OE13 transgenic lines produced fewer productive panicles than the wild-type ZH11, while the OE5 line did not exhibit such a reduction. In all three transgenic lines, the number of grains per panicle and 1000-grain weight remained unchanged. However, compared with ZH11, all *DTX6m*-overexpression lines had altered seed morphology, including grains with increased length, unchanged width, and reduced thickness. Additionally, no significant differences in starch or total protein content were detected between the transgenic and wild-type plants. Overall, among the transgenic lines, OE5 exhibited the most superior performance, demonstrating robust resistance to paraquat and diquat while maintaining rice yield and nutritional quality. These findings highlight the critical need for screening large transgenic populations to identify optimally performing lines for agricultural implementation. The significantly elevated accumulation of free amino acids in all three independent *DTX6m*-OE lines (Figure 7B) implies a potential role for DTX6 in amino acid homeostasis. However, the precise function of DTX6 in *Arabidopsis* still remains elusive at present. Future research will focus on identifying the endogenous substrates of DTX6 to clarify its physiological role. In addition, introducing *DTX6m* into diverse rice cultivars across distinct geographical regions and evaluating the agronomic traits of DTX6m-transgenic lines under variable environmental conditions would facilitate a comprehensive systematic evaluation of the gene’s functional performance. The lack of comprehensive testing of *DTX6m* across diverse rice genetic backgrounds and dynamic environmental conditions represents a significant limitation of the present study, which necessitates further research.

## 4. Materials and Methods

### 4.1. Rice Variety and Growth Conditions

All rice materials used in this study were of *Oryza sativa* L. cv. Zhonghua 11 (ZH11) background. For the greenhouse assays, they were grown under a 12 h light (30 °C)/12 h dark (22 °C) photoperiod with 50% humidity unless otherwise specified. For the field assay, rice seeds were germinated and grown in the green house for three weeks and then transplanted in June to the paddy field within the rice transgene experimental base located at Fudan university campus. They were harvested in October.

### 4.2. Generation of Transgenic Lines

The open reading frames of *DTX6*-*G311E* were cloned into the pCAMBIA1301-N-eYFP vector by KpnI/BamHI sites. The construct was transformed into rice using the *Agrobacterium tumefaciens*-mediated transformation method [43], with assistance provided by EDGENE Biotechnology Co., Ltd. (Wuhan, China). The primers used in clone construction for overexpression are listed in Supplementary Table S1.

### 4.3. RNA Extraction and RT-qPCR

Total RNA was extracted using TRIzol reagent (TaKaRa, Shiga, Japan) and reverse-transcribed with PrimeScript RT reagent (TaKaRa, Shiga, Japan). qPCR was performed with a SYBR Premix Ex Taq kit (TaKaRa, Shiga, Japan) using a CFX96 real-time PCR detection system (Bio-Rad, Hercules, CA, USA). The rice *UBQ5* gene was used as an internal control to normalize different samples. RT-qPCR primer sequences are given in Supplementary Table S1.

### 4.4. Herbicide Spraying Assay of Soil-Grown Seedlings

For the paraquat resistance assay in the greenhouse, the homozygous overexpression lines and ZH11 wild type were grown in soil for one month, and then sprayed with 0.5 g/L mM paraquat. Then, 10 days after spraying, the survival rates and wilted leaves of different lines were documented and plants were photographed. For the diquat resistance assay, plants were grown for 25 days and then sprayed with 0, 0.5, 1, 2, and 5 g/L diquat. Then, 3 days post-application, the phenotypes were recorded and the survival rate was calculated. For the field tests, the overexpression lines and ZH11 wild type were grown side-by-side for two months till the reproductive stage; then, 2 g/L diquat was sprayed and pictures were taken after 10 days.

### 4.5. Agronomic Trait Analysis of Transgenic Rice Plants

Rice plants were grown from June to October in the paddy field within the rice transgene experimental base located at Fudan university campus. At least 12 plants of each line were randomly chosen in the field for further agronomic trait analysis, including plant height, flowering time, effective panicle number per plant, grain number per panicle, panicle length, one-thousand-grain weight, and grain size. One-thousand-grain weight was measured using harvested seeds that were air-dried for two weeks. Grain size including length and width was analyzed using an automatic seed-examining high-speed document camera (Shenzhen Liangtian, Shenzhen, China) and the thickness was measured manually with vernier calipers.

### 4.6. Nutritional Components of Rice Seeds

About 2 g dehulled rice seeds were ground into fine powder using a grinding mill and then used for the following determination of nutritional components.

#### 4.6.1. Free Fatty Acids

Free fatty acids (FFAs) were extracted and quantified according to the established protocol with modifications [44,45]. Briefly, 0.1 g of rice powder was homogenized in 1 mL of chloroform–heptane–methanol (200:150:7, *v*/*v*/*v*) containing silicic acid (0.05 g/mL) to eliminate phospholipids. The mixture was agitated for 3 h at room temperature, followed by centrifugation at 8000× *g*, 4 °C for 10 min. The supernatant was collected and reacted with freshly prepared Cu-TEA solution [0.05 M Cu(NO_3_)_2_, 0.1 M triethanolamine, NaCl-saturated (~33 g/100 mL), pH 8.1] to form FFA-Cu complexes. The FFA content was then determined using a commercial Free Fatty Acid Determination Kit (Suzhou Keming Co., Ltd., Suzhou, China).

#### 4.6.2. Total Sugar

Total sugar content was determined using the 3,5-dinitrosalicylic acid (DNS) method [46]. Briefly, 0.1 g of rice powder was homogenized in 1.5 mL of ddH_2_O, followed by the addition of 1 mL of 6 N HCl. The mixture was incubated in a 95 °C water bath for 30 min to hydrolyze polysaccharides. After cooling to room temperature, the sample was neutralized with ~1 mL of 6 N NaOH, diluted to a final volume of 10 mL with ddH_2_O, and centrifuged at 8000× *g* for 10 min at 25 °C. The supernatant was then analyzed for sugar content using a Total Sugar Determination Kit (Suzhou Keming Co., Ltd., Suzhou, China) according to the manufacturer’s instructions.

#### 4.6.3. Soluble Sugar

Soluble sugar content was determined using the anthrone colorimetric method [47] with a Plant Soluble Sugar Determination Kit (Suzhou Keming Co., Ltd., Suzhou, China). The soluble sugar was extracted from 0.1 g of rice powder with 1 mL of distilled water at 95 °C for 10 min. After cooling, samples were centrifuged at 8000× *g* for 10 min at 25 °C. The supernatant was collected and diluted to 10 mL with ddH_2_O, and 40 µL aliquots were used for soluble sugar quantification according to the manufacturer’s protocol.

#### 4.6.4. Starch Content

Starch was extracted and determined as described previously [48] with some modifications. For this purpose, 0.1 g rice powder was extracted with 1 mL of 80% ethanol at 80 °C for 30 min. After centrifugation at 3000× *g* for 5 min, the supernatant was discarded and the pellet was air-dried. The air-dried residue was resuspended in 0.5 mL ddH_2_O, heated in a boiling water bath for 15 min, and cooled. For starch extraction, 0.35 mL of 9.2 M perchloric acid was added and the mixture was incubated for 15 min at room temperature. The solution was then diluted with 0.85 mL ddH_2_O and centrifuged at 3000× *g* for 10 min. The resulting supernatant was analyzed for starch content using a Plant Starch Content Determination Kit (Suzhou Keming Co., Ltd., Suzhou, China) according to the manufacturer’s instructions.

#### 4.6.5. Protein Levels

Next, 0.02 g of rice powder was subjected to sequential extractions to obtain the four major rice storage proteins (albumin, globulin, prolamin, and glutelin) according to the method described previously [49]. The contents of different protein ingredients were determined using a Modified Bradford Protein Assay Kit (Sangon Biotech, Shanghai, China).

#### 4.6.6. Free Amino Acid Levels 

0.1 g rice powder was extracted with 1 mL of distilled water in a boiling water bath for 15 min. After cooled to room temperature, samples were centrifuged at 10,000× *g* for 10 min. The supernatant was used for total amino acid determination via the ninhydrin method [50].

### 4.7. Phylogenetic Analysis

The amino acid sequences of *Arabidopsis* and rice MATE family proteins were extracted from the TAIR (https://www.arabidopsis.org/, accessed on 25 June 2025) and RAP-DB (https://rapdb.dna.affrc.go.jp/, accessed on 25 June 2025) websites. In total, 56 *Arabidopsis* MATE family proteins and 46 rice MATE proteins (for genes with multiple transcripts, only the first transcript was chosen) were analyzed for their phylogenetic relationships using *Selaginella_moellendorffii* as the alien species. Multiple sequence alignment was performed with Clustal X using default parameters. The resulting alignment was output in Phylip format and uploaded to the online PhyML (http://www.atgc-montpellier.fr/, accessed on 25 June 2025) website to construct the phylogenetic tree using the maximal likelihood method by standard bootstrap analysis with 1000 replicates [51]. The resulting phylogenetic tree was visualized using the iTOL tool (https://itol.embl.de, accessed on 25 June 2025).

## 5. Conclusions

Our results reveal that the *DTX6m* gene effectively confers resistance to paraquat and diquat in rice without compromising yield potential. Moreover, its overexpression leads to early flowering, semi-dwarfism, and increased free amino acid levels in rice grains. The phenotypes induced by DTX6m in rice suggest generalizability to other monocot cereal crops. Collectively, our findings in rice and previous results for *Arabidopsis* indicate that DTX6m holds broad utility for improving herbicide resistance in both monocot and dicot plants.

## Data Availability

Data will be made available on request.

## Supplementary Materials

The following supporting information can be downloaded at: https://www.mdpi.com/article/10.3390/ijms26136204/s1.
