# Supplementary material for: The G311E Mutant Gene of MATE Family Protein DTX6 Confers Diquat and Paraquat Resistance in Rice Without Yield or Nutritional Penalties
Source: Int J Mol Sci. 2025 Jun 27;26(13):6204. doi: 10.3390/ijms26136204 (PMC12249753; doi:10.3390/ijms26136204)
Supplement: Supplementary file 1 [file ijms-26-06204-s001.zip › ijms-3682986-supplementary.pdf]

Supplementary Figures and Tables

Figure S1

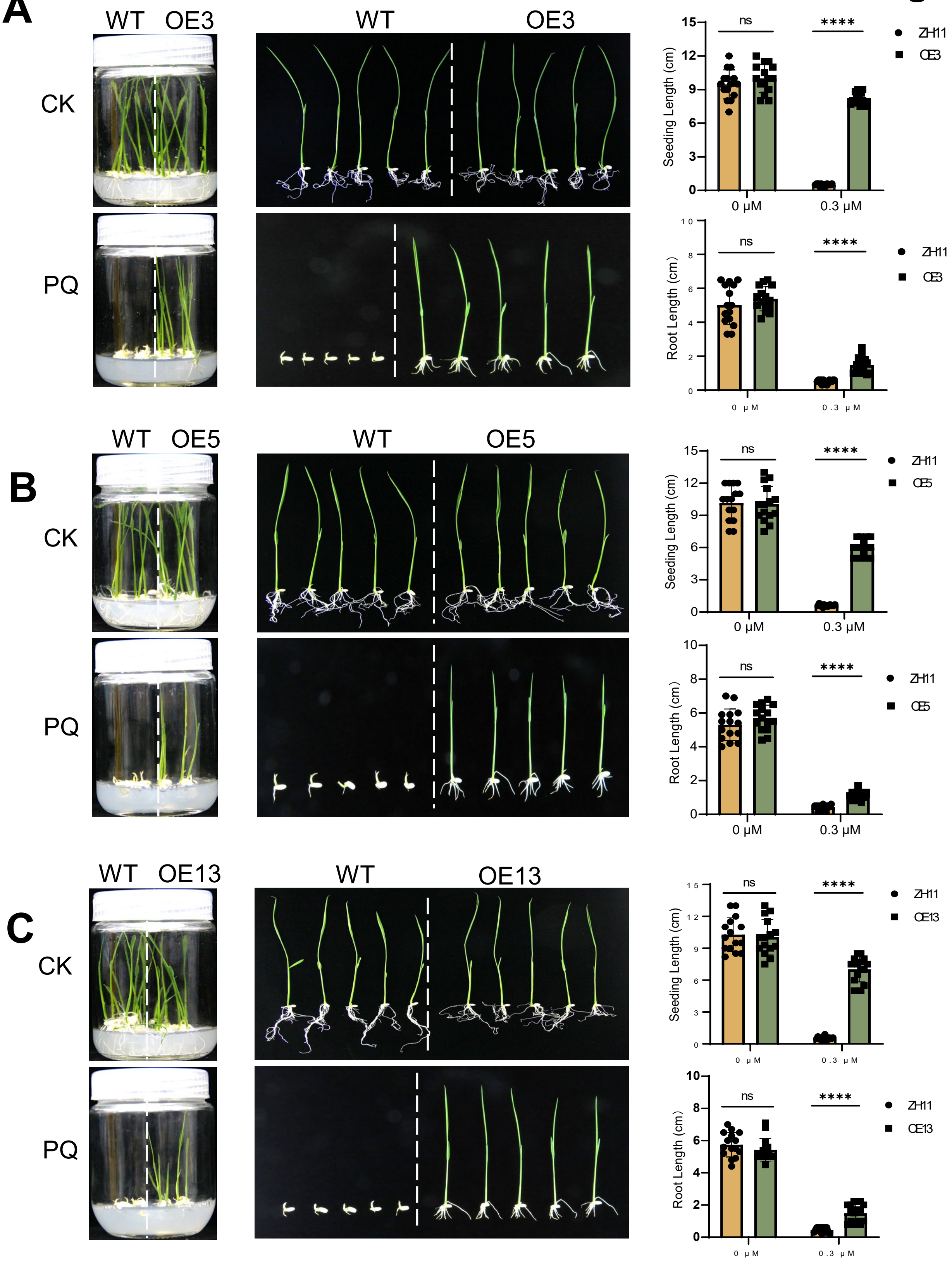

FigureS1. Leaf and root phenotypes of three *DTX6m*-overexpression lines on ½ MS media containing paraquat. Rice seeds were dehulled, sterilized and germinated on ½ MS media supplemented with or without 0.3 μM paraquat for 8 days, then the root and seedling length were measured. Data represent means ± SD of 15 seedlings. Student's t-test was performed to analyze the significance of difference between ZH11 and *DTXm*-overexpression lines. **** $P \leq 0.0001$.

Figure S2

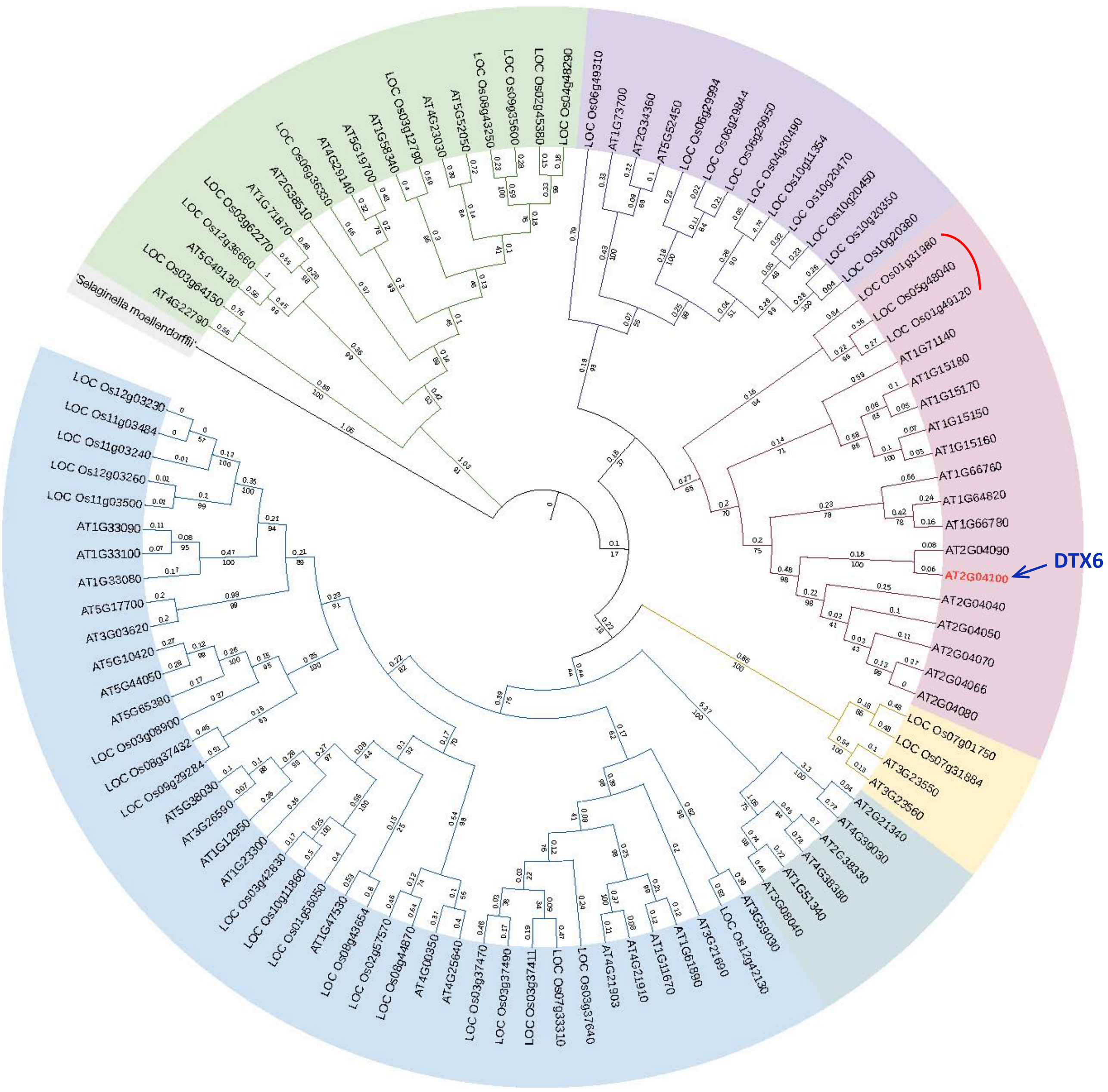

Figure S2. Phyolgenetic tree of DTX6 homologs in rice and *Arabidopsis.* The phylogenetic tree was drawn by PhyML(http://www.atgc-montpellier.fr/) with the Maximum Likelihood method. Different groups are marked with different colors. The MATE homolgous gene in *selaginella moellendorffii* was used as the alien species. DTX6 and three rice MATE proteins with the closest evolutionary proximity to DTX6 are marked.

Figure S3

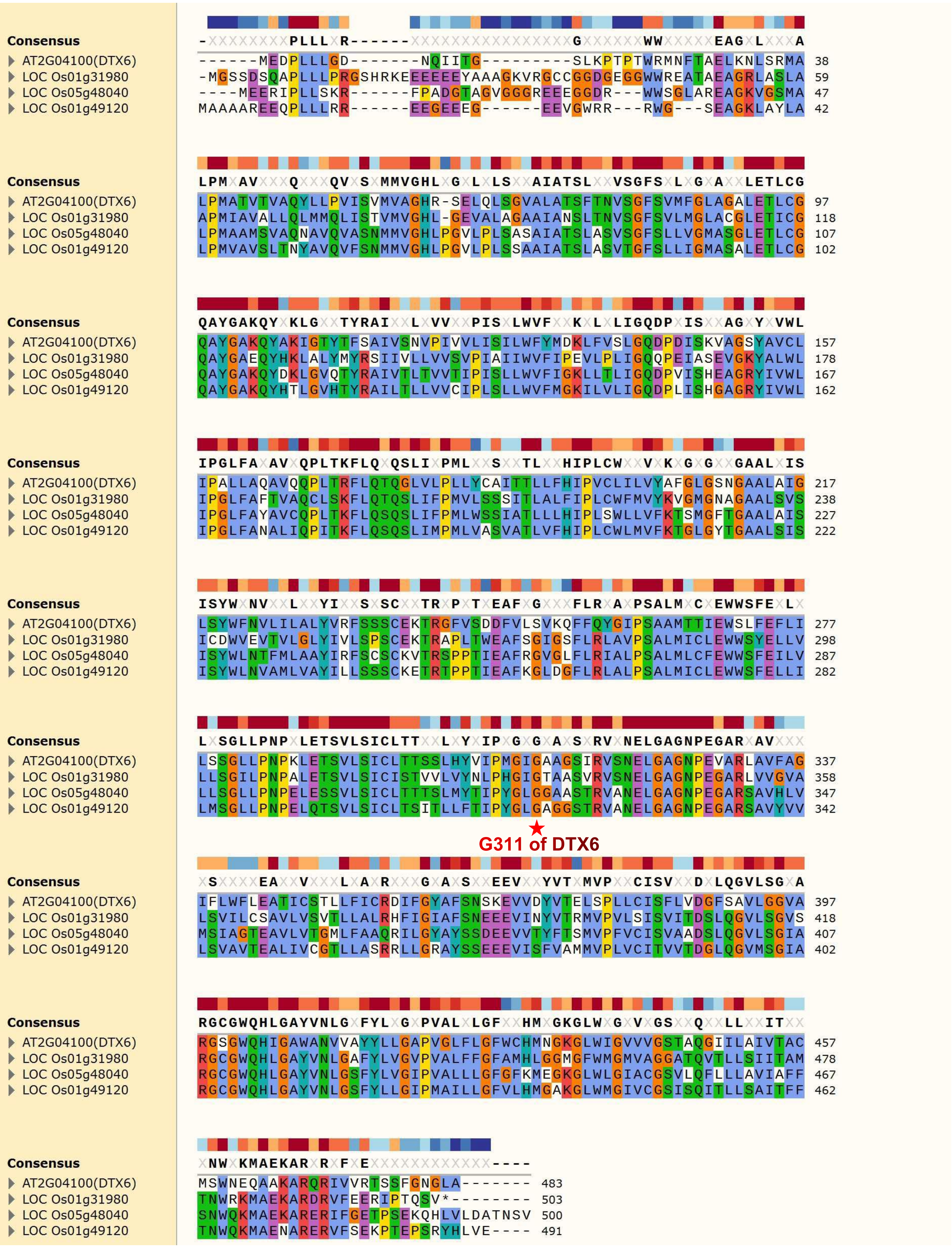

FigureS3. Amino acid sequence alignment of *Arabidopsis* DTX6 and the three most similar rice MATE family members. The G311 residue of DTX6 is marked by a star in the figure.

Supplemental Table S1

Table S1. Primers used in this study

Primer name	Primer sequence
**OE vector construction**	
eYFP-DTX6m-F	CGGGGTACCATGGAAGATCCACTTTTATTGGG
eYFP-DTX6m-R	CGCGGATCCTCAAGCAAGTCCATTGCCAA
**RT-qPCR**	
UBQ5-FP	AATGTGAAGGCGAAGATCCAAGAC
UBQ5-RP	AGACGGAGGACGAGATGAAGC
DTX6M-F	GACAATCAGATAATCACCGGAAG
DTX6m-R	ATTGAGCAACAGTCACGGTGG
